# Investigation of a Short Carbon Fibre-Reinforced Polyamide and Comparison of Two Manufacturing Processes: Fused Deposition Modelling (FDM) and Polymer Injection Moulding (PIM)

**DOI:** 10.3390/ma13030672

**Published:** 2020-02-03

**Authors:** Elena Verdejo de Toro, Juana Coello Sobrino, Alberto Martínez Martínez, Valentín Miguel Eguía, Jorge Ayllón Pérez

**Affiliations:** 1 Faculty of Industrial Engineering, 02071 Albacete, Spain; elena.verdejo@alu.uclm.es (E.V.d.T.); valentin.miguel@uclm.es (V.M.E.); 2 Materials Science and Engineering, Instituto de Desarrollo Regional, 02071 Albacete, Spain; alberto.martinez@uclm.es (A.M.M.); jorge.ayllon@uclm.es (J.A.P.)

**Keywords:** additive manufacturing, fused deposition modelling, composites, 3D printing, polymer injection moulding, polyamide, CFRP

## Abstract

New technologies are offering progressively more effective alternatives to traditional ones. Additive Manufacturing (AM) is gaining importance in fields related to design, manufacturing, engineering and medicine, especially in applications which require complex geometries. Fused Deposition Modelling (FDM) is framed within AM as a technology in which, due to their layer-by-layer deposition, thermoplastic polymers are used for manufacturing parts with a high degree of accuracy and minimum material waste during the process. The traditional technology corresponding to FDM is Polymer Injection Moulding, in which polymeric pellets are injected by pressure into a mould using the required geometry. The increasing use of PA6 in Additive Manufacturing makes it necessary to study the possibility of replacing certain parts manufactured by injection moulding with those created using FDM. In this work, PA6 was selected due to its higher mechanical properties in comparison with PA12. Moreover, its higher melting point has been a limitation for 3D printing technology, and a further study of composites made of PA6 using 3D printing processes is needed. Nevertheless, analysis of the mechanical response of standardised samples and the influence of the manufacturing process on the polyamide’s mechanical properties needs to be carried out. In this work, a comparative study between the two processes was conducted, and conclusions were drawn from an engineering perspective.

## 1. Introduction

Fused Deposition Modelling (FDM) is a promising technology framed within Additive Manufacturing (AM) and has been implemented in the manufacturing processes of functional structures with complex geometries, showing interesting responses in different fields [1]. Medicine, biotechnology, automotive industry or aerospace are domains that use such technologies to obtain parts made of polymers or polymer–matrix composites with high stiffness and low weight values, also reducing material waste during the process [2]. Among the different technologies that are part of AM, those expected to be most suited to the manufacturing industry are Selective Laser Sintering (SLS), Selective Laser Melting (SLM) and FDM [3]. The last of these technologies is considered to be more developed than the other two [4]. FDM technology consists of the layer-by-layer deposition of a thermoplastic polymer previously driven into a semi-liquid state through the nozzle that allows the deposition of the material. The most widely processed polymers are acrylonitrile butadiene styrene (ABS), polylactic acid (PLA), polyvinyl alcohol (PVA), polyamides (PA) or polyether ether ketone (PEEK) [5]. Three-dimensional printing is also considered a sustainable production technology, which means that material waste is reduced in the manufacturing process, or even avoided [6]. In addition, the high geometrical accuracy in the dimensions of the printed parts is also a determining factor [7]. Design flexibility is considered an advantage of the FDM process as it takes into account not only the idea of modifying the prototype as much as desired, but also considers the wide variety of parameters that this technology allows to be used [8]. Infill density, infill pattern, layer height or printing velocity are the parameters traditionally considered [9]. Nevertheless, other aspects that are sometimes neglected might have an influence on the part being manufactured [10]. For example, Chacón et al. demonstrated the influence of the raster angle and the placement of the part and established the optimum location to obtain the best properties, studying the behaviour of parts under tensile and bending loads [11]. It is necessary to study the influence of these parameters on the mechanical response of printed parts to create a summary that finds the best combination for each application, depending on different stress types [12]. Some authors have also studied the anisotropy of AM parts, determining the significant effect of the placement of the part on the build plate [13].

The main disadvantages of printed parts that need to be addressed include the poor mechanical properties limited by the polymeric matrix and the poor adhesion between consecutive layers during deposition [14]. Some works have studied the influence of parameters on polymers, such as PA12 (ultimate tensile strength (UTS) = 33 MPa) or reinforced PA12 [15]. However, the mechanical properties of PA12 are lower than PA6 (UTS higher than 50 MPa), which has been considered a more suitable choice for replacing metal parts [16]. Higher stiffness and strength values are obtained in PA6 than in PA12, which is more widely used in FDM research, considering the limitations on the melting temperature according to the thermal resistance of printed parts [17]. Other materials, such as ABS and PLA, have been compared to PA, but have yielded worse results [18]. Nylon 6 showed a tensile strength of ~80 MPa vs. ~50 MPa for PLA and ~65 MPa for ABS considering injected parts. In the case of AM manufactured parts, lower results were obtained: a tensile strength of ~50 MPa for nylon 6, ~45 MPa for PLA and ~25 MPa for ABS [18].

Alone, matrices do not reach the expected requirements in mechanical behaviour. To solve the low strength and stiffness problem, the use of reinforcement has been considered the best alternative [19]. Carbon and glass fibres (short or continuous) and nanoparticles, such as nanospheres, are used to reinforce the polymeric matrix [20]. For reinforced polyamide, Chabaud et al. obtained mechanical properties similar to aluminium alloys [21]. Carbon fibres have been chosen as a favourite reinforcement due to their high stiffness and strength, and low weight [9,21]. 

Despite having shown higher mechanical properties as a matrix in composites, printed parts made of short carbon fibre-reinforced PA6 have not been fully studied. Furthermore, previous research has focused on evaluating the tensile or dynamic properties but has not considered the compressive properties. Moreover, knowledge is needed about which manufacturing technique fits better with the requirements of parts. In addition, some new technologies, such as FDM, must be compared with traditional ones, in this case polymer injection moulding, to determine whether the latter might offer a more effective solution. Taking into account all the above-mentioned considerations, this experimental work focuses on evaluating and comparing the response of printed and injected samples, while also considering the possibility of dealing with compressive loads. The combination, or even the replacement of traditional technologies with new ones, will be essential for the manufacturing process of different functional parts in forthcoming years. This work presents this comparison and provides results for different mechanical tests summarised from an engineering perspective.

## 2. Materials and Methods 

### 2.1. Raw Material and Equipment

The short carbon-fibre-reinforced material used as feedstock in the printing process was CarbonXTM CRF-Nylon (3DX Tech, Grand Rapids, MI, USA), a filament of PA6 with a diameter of 2.85 mm. Fibre content was 20 weight percent (wt %). Furthermore, an Olsson Ruby nozzle of 0.4 mm replaced the printer’s default brass nozzle as it has previously been proven that fibre damages brass after a few printed samples. The printer was an Ultimaker 2 Extended + (Ultimaker, Utrecht, Netherlands). Samples were designed using Autodesk Inventor (2016 version, Autodesk, St. Raphael CA, USA), and the slicing program was Cura 3.5.1 (Ultimaker, Utrecht, The Netherlands). In the case of the injection moulding, the same material was used but in granulated form. Although the granulate could have been obtained directly from the filament, we decided to use the granulated composite from the same supplier in order to work with a homogeneous size of grain. 

### 2.2. Parameters for Manufacturing

As our aim was to study the differences in the behaviour of samples manufactured by injection moulding and 3D-printing technologies, we needed to distinguish and set different parameter types corresponding to each manufacturing technique.

**Figure 1:**
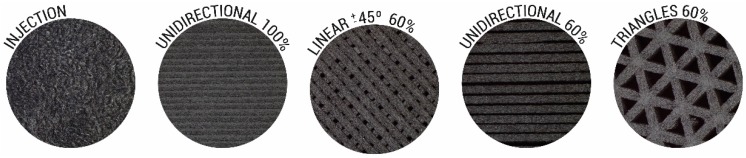
Stereomicroscope images (×1.25) of the appearance of the injected and different patterned printed samples.

Three-dimensional-printing parameters were set following the recommendations of the feedstock manufacturer. The nozzle temperature was 260 °C, the build plate temperature was 80 °C, the printing speed was 50 mm/s and the layer height and nozzle diameter were 0.1 mm and 0.4 mm, respectively. In order to compare the samples manufactured by injection moulding, we manufactured samples with 100% infill density using the longitudinal pattern. The 60% infill density parts were manufactured by employing different patterns (triangles, lines ±45° and longitudinal; see Figure 1) to compare them in bending, compression and tensile tests. 

The mould temperature was set at 80 °C, whereas the polymer temperature during injection was 260 °C. The pressure in the process was 8.5 bar. Injection moulding parameters were chosen to be as close as possible to those used in the printing process. 

### 2.3. Measurement Techniques

**Figure 2:**
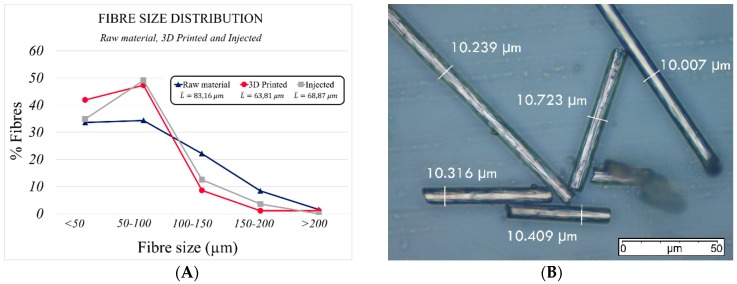
Results of the fibre length distribution in the raw material, injected and printed samples (**A**) and measurement of diameters in fibres using 400× with a microscope (**B**).

The influence of both processes on fibre length was first investigated. The specimens manufactured by both 3D printing and injection moulding were extracted and burnt in Thermogravimetry-Differential Thermal Analysis (TG-DTA) equipment (EXSTAR 6200, Seiko Instruments, Chiba, Japan). The process started at 23 °C and went to 900 °C in an inert nitrogen atmosphere to avoid fibre degradation. The heating rate was 20 °C/min at an inert gas flow of 200 mL/min. Having obtained fibres, measurements were taken with a microscope. Three specimens of each manufactured sample were measured, obtaining the results shown in Figure 2. 

In many experimental studies, the printer’s thermal environment affects the degree of crystallinity of the composite’s polymeric matrix, which strongly influences its mechanical properties. In general, the higher the degree of crystallinity, the greater is the strength and the less is the deformation generated in mechanical tests. To obtain reliable results in mechanical tests, a previous analysis of the thermal environment in the printing process was carried out. To this end, three build plate temperatures were chosen in order to determine whether the thermal environment had an impact on the mechanical response of printed parts: 110 °C, 60 °C and 25 °C, which was considered representative of the room condition. DSC tests of samples after printing were conducted and bending tests were carried out to study whether crystallinity appears in the material and the response of the printed parts to mechanical stresses. Although in all three cases, the build plate temperature was lower than the initial characteristic crystallisation temperature determined for the material, the gradient of temperature from the printing temperature (260 °C) to the temperature set for the build plate was found to promote crystallinity. 

All mechanical tests were conducted under room conditions (23 °C, HR 50%). The tensile properties of the samples were obtained according to UNE EN ISO 527-2, with a Zwick Z010 TN2S using an extensometer also produced by Zwick with a calibrated length (L0) of 25 mm. Data were collected and processed with the testXpert machine software (8.1 version, ZwickRoell, Ulm, Germany). The distance between grips was 115 mm, and test velocity was set at 1 mm/min. 

A compression test was conducted as standard, but recommendations were taken from UNE EN ISO 14126:2001. The cylindrical samples (ϕs = 12 mm, Ls = 18 mm) were manufactured by both injection moulding and 3D-printing. To do so, a new steel mould with the sample’s chosen dimensions had to be manufactured according to the requirements of the above-mentioned injection equipment. Compression velocity was also set at 1 mm/min. Two flat carbon steel discs (ϕ = 16 mm) were used to compress the sample. The same Servosis machine as in the bending test was used to carry out the test, following UNE EN ISO 178:2011. Molybdenum (IV) sulphide was placed on the surface of the flat discs to reduce the friction between samples and discs, and to prevent the sample from becoming barrel-shaped during the test.

The moulds for injection were designed with only one 6 mm gate; no runners were used in order to obtain a direct material flow into the cavity. The gate was placed at one end of the part to be obtained, which in the worst case was 160 mm (tensile specimens). These designs were elaborated to make the flow of the material easier in spite of the maximum available pressure of the equipment.

## 3. Results and Discussion

### 3.1. Fibre Length Analysis 

After the calcination process in an inert atmosphere, a hundred fibres of each specimen were obtained and measured. The results of the histograms of the lengths of the initial filament (raw material), the 3D printed and the injected material, are shown in Figure 2A. Both processes had a negative influence on fibre length. The average initial feedstock filament length was $\bar{L}=83.16\;\mu m.$ However, after printing and injection moulding, fibre length decreased by up to 23.27%. 

This may have a number of consequences on the behaviour of the manufactured samples if the critical length ($L_{c}$) is greater than the fibre length of the processed parts, since the reinforcing effect is considered to be less effective. In order to obtain the critical fibre length, it was necessary to determine the tensile strength of the fibre ($\sigma_{f}$), its diameter ($\phi_{f})$ and the interfacial shear strength (IFSS) fibre-matrix. This last property can only be found by performing a pull-out test of the composite material. Once the necessary parameters were identified, the critical length was obtained by applying Equation (1) [16].

(1) $$L_{c}=\frac{\sigma_{f}\cdot \phi_{f}}{2\cdot IFSS}$$

The value of $\sigma_{f}=2.2\;\mathrm{GN}\cdot m^{-2}$ was taken from the literature [16]. The experimental measurement of the diameter of the fibre was found to be $\phi_{f}=10.12\pm 0.94\;\mu m$ (Figure 2B). The shear stress of the interfacial bonding between the carbon fibre and the matrix (IFSS=44 MPa) was taken from experimental results reported in different research works [1,22,23] because it was not possible to run the pull-out test in this experimental study. Carbon fibres were considered to be unsized.

The critical length obtained by Equation (1) was $L_{c}=253\;\mu m$. Therefore, as the length of the fibres inside the matrix was shorter than the critical theoretical value, the reinforcing effect would be lower than for a fibre length longer than the critical one, especially in the tensile test. Nevertheless, fibres with a longer length than the critical one were observed after both manufacturing processes; that is, 3D-printing and injection moulding. As a result, the reinforcing effect would take place, but to a lower extent and only as a result of the longest fibres. Moreover, some researchers [9] have found that the 3D-printing process reveals a greater influence on the mechanical behaviour of the part than the reinforcement effect itself. In addition, some studies report that the highest reinforcing volumes can only be reached by using short fibres and taking into account that in AM, the mixing–printing process itself tends to result in a length shorter than the critical one [17].

### 3.2. Study of Variation in Crystallinity in the Printed Samples

**Figure 3:**
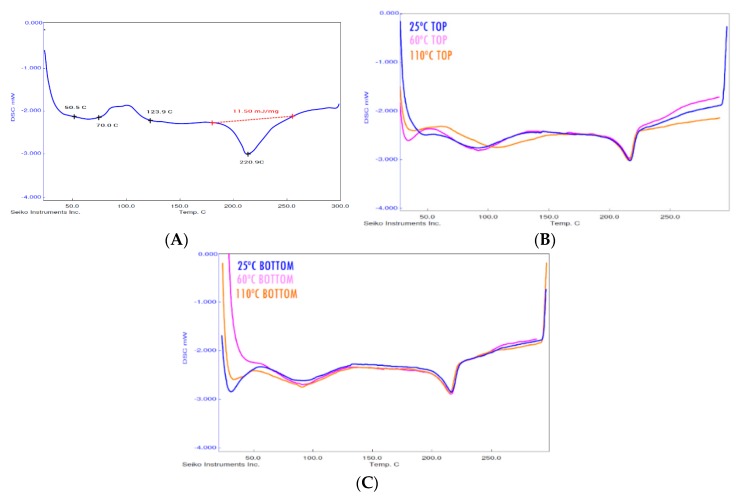
The DSC analysis of the samples printed at different built plate temperatures to analyse the influence of the thermal environment on the degree of crystallinity. The DSC analysis of raw material is shown in (**A**), the top parts are the analyses shown in (**B**) and the bottom parts are the analyses shown in (**C**).

The DSC tests were carried out with the three printed samples of each type (at different build plate temperatures). The results showed no variation in crystallisation peak, starting point or ending point. The samples behaved the same in the thermal analysis and the results obtained were satisfactory, as shown in Figure 3B,C.

DSC analysis of the raw material, i.e., the CarbonXTM CRF-Nylon wire, showed a crystallisation peak (Figure 3A), whereas this was not seen in the DSC analysis carried out for 3D printed parts (Figure 3B,C) independently. For this reason, it can be concluded that the AM manufacturing technique promotes the crystallinity of the PA6 matrix. 

**Figure 4:**
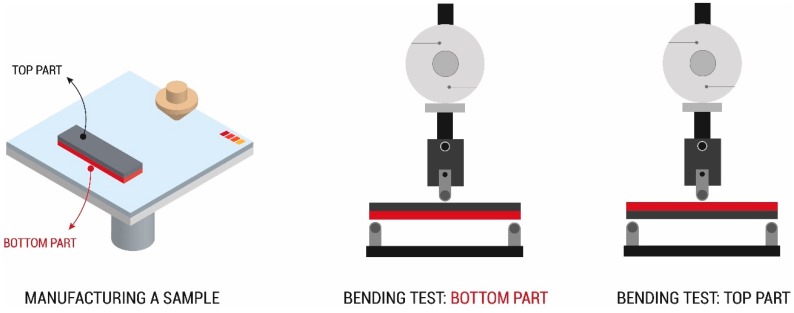
Positioning samples in the bending test, labelled (top and bottom) according to printing placement.

There was no variation between the top and bottom parts of the same sample, which were those farthest from and closest to the build plate, respectively (Figure 4). This result is also supported by Figure 3 showing that the DSC curves corresponding to bottom and top surfaces are identical; the initial parts of the curves are typically different according to the initial humidity conditions of the samples and/or the stabilisation time of the furnace chamber. In the bending test, the top and bottom parts in the same samples were tested with bending stresses as a fracture appeared in the stretched part. Both parts were tested to check the influence of the thermal gradient during the printing process.

**Figure 5:**
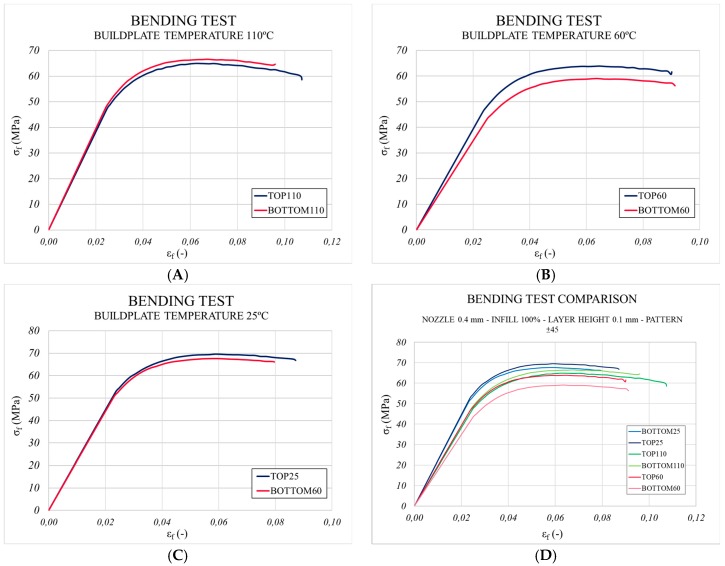
Analysis of the mechanical response of the samples manufactured by 3D printing at different build plate temperatures to study if anisotropy was caused by the thermal environment. (**A**) Build plate temperature: 110 °C, (**B**) build plate temperature: 60 °C, (**C**) build plate temperature: 25 °C and (**D**) bending test comparison of (**A**–**C**).

The results obtained for the maximum flexural strength ($\sigma_{fM}$), Young’s Modulus ($E_{f}$) and yield strength ($Y_{f}$) showed a negligible variation between tested specimens (Table 1) and, consequently a negligible influence of the build plate temperature on the flexural behaviour of samples (see Figure 5). The degree of crystallinity was not affected by the thermal gradient, which appeared in the manufacturing process under the set conditions.

Homogeneous behaviour due to the uniformity of each sample regarding its crystallinity was expected. Therefore, the mechanical properties of parts would be independent of the thermal gradient appearing in the 3D printing process and the build plate temperature would not be considered a variable affecting the part.

### 3.3. Determination of Properties in the Tensile Test

**Figure 6:**
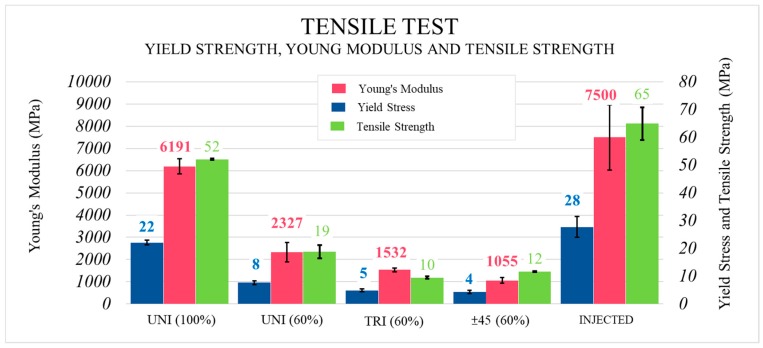
Tensile test. Results obtained for Young’s Modulus, yield strength and tensile strength of the injected and 3D printed samples.

The tensile test results showed marked differences between the 60% filled parts and the 100% filled ones. As expected, infill density had a significant impact on the three mechanical parameters under study (see Figure 6). Ultimate tensile strength (UTS) was higher when the completely filled unidirectional pattern was used, which allowed 63% more stress than the 60% filled one. The results obtained for Young’s Modulus showed improvements of up to 62% for the completely filled samples. For yield stress, the same occurred as in tensile strength.

When studying the influence of pattern, the unidirectional pattern behaved better than the triangular one (improvements of 47%) and the linear ±45° (up to 37%). For yield stress and Young’s Modulus, the same occurred between hollow patterns. Due to the non-deformability of the triangles in the triangular pattern, the samples built using that distribution of filaments displayed greater levels of stiffness, which might be due to a higher Young’s Modulus compared to the alternative linear ±45° pattern (30% higher in the former). Nevertheless, the best result was obtained with the unidirectional pattern (improvements up to 55%) due to filament orientation according to the preferable distribution of stress in the axial direction in the tensile test. However, the ±45° pattern is used in applications where the direction of the stresses is unknown or known but non-axial, whereas the unidirectional pattern might have a worse response to the mechanical stresses loading the part. 

Consequently, not only infill density but also the infill pattern was a determining factor in the results obtained. 

**Figure 7:**
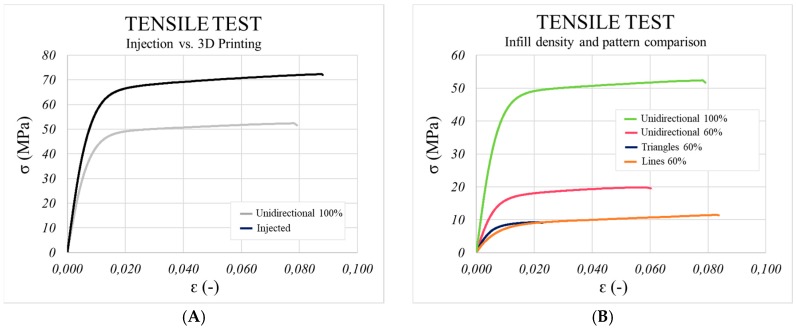
Comparison of the behaviour of the injected and printed samples with stretching stresses in the tensile test (**A**), and the influence of infill density and printing pattern on the mechanical behaviour of the printed parts (**B**).

When comparing the printed and injected parts (Figure 7), it can immediately be seen that the injected samples behaved better under axial stresses. However, differences were not as large as expected. Yield stress was 21% higher, whereas Young’s Modulus was 17% greater and tensile strength at the break point was only 20% higher in the injected samples. Comparing FDM and IM, Lay et al. obtained similar results [18], while Blok. et al. tested a printed short carbon fibre-reinforced nylon (6 wt % of fibre weight content) and reported a UTS = 33 MPa and E = 1900 MPa [9]. In the case of the current study, for the unidirectional pattern, UTS = 52 MPa and E = 6191 MPa, but the percentage of fibre inside the polymeric matrix is also higher (20 wt %). Furthermore, greater deviations from the average were obtained with the injected samples, in which more homogeneous behaviour was expected than for the 3D printed samples. It can be concluded that the printed parts would be suitable to replace the injected ones when working under stretching loads.

**Figure 8:**
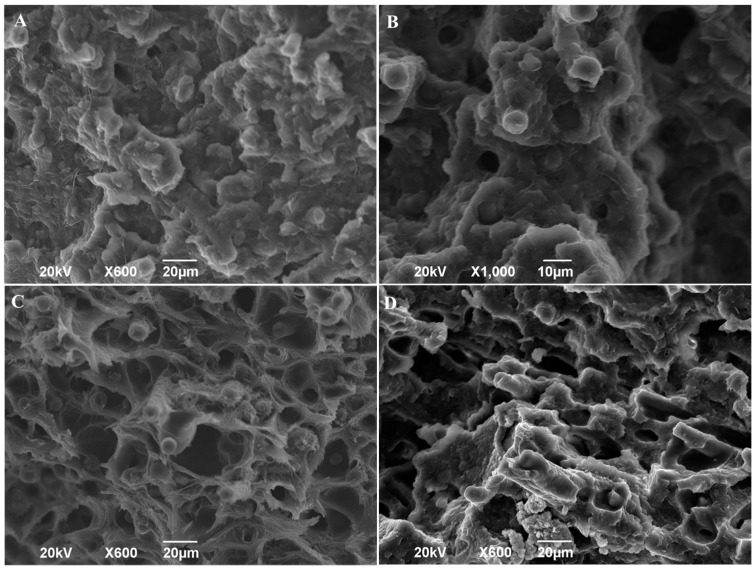
Fractographies of tested tensile samples: (**A**) Injection moulding 600×; (**B**) injection moulding 1000×; (**C**) 3D printed unidirectional 0° 600×; (**D**) 3D printed ±45° 600×.

From the fractographies in Figure 8, a sound structure for the parts obtained by injection moulding (Figure 8A) can be observed. Properly, the fractography is dominated by the matrix and fibres fracture, and to some extent, by the fracture of the matrix–fibre interface. This reveals that the grade of polymerisation obtained in the injection moulding process is reasonable despite the low-pressure injection condition (8.5 bar). The fractures of the samples obtained by 3D printing show that pores and fibres have been separated from the matrix interface in a greater proportion (Figure 8C,D). This is a consequence of the short length fibre used leading to an insufficient interface area with the PA6 matrix. From this viewpoint, the interface is more suited to the injection moulding samples.

### 3.4. Determination of Properties in the Compression Test

**Figure 9:**
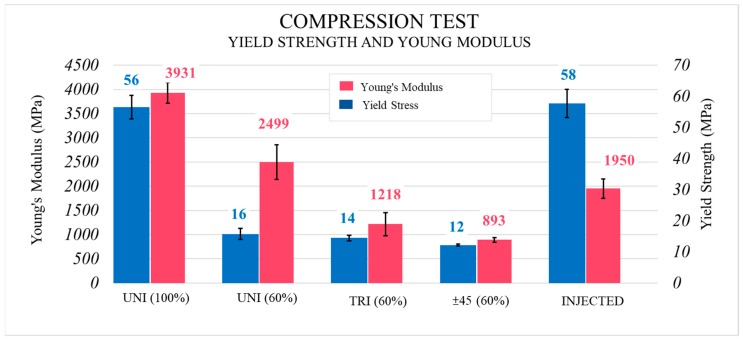
Compression test. Results obtained for yield strength, tensile strength and Young’s Modulus of the injected and 3D printed samples.

**Figure 10:**
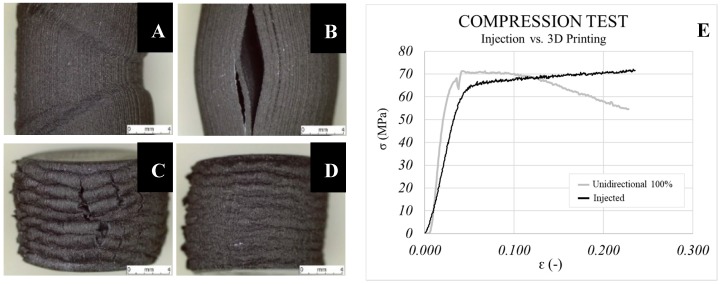
Fracture images of samples under compressive stress. (**A**) Unidirectional 100%, (**B**) unidirectional 60%, (**C**) triangles 60% and (**D**) linear ±45. (**E**) Comparison between the injected and printed samples in the compression test.

**Figure 11:**
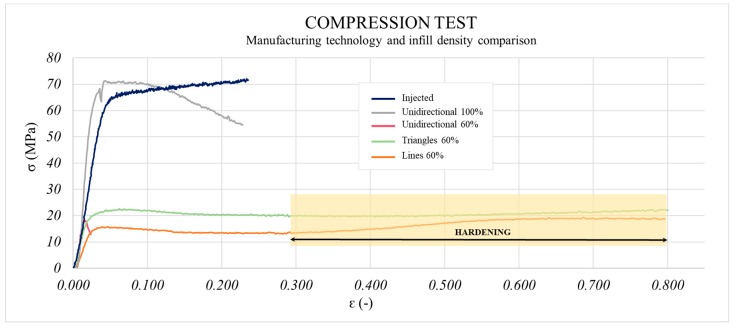
Compression test: influence of pattern on the behaviour of sample under compressive stresses.

The compression test results are shown in Figure 9 and indicate improvements of only 4% in the injected samples in comparison with the 3D printed ones (100% infill). It is worth noting that Young’s Modulus is higher in the printed parts (50% improvement with respect to the injected samples). This suggests that when compressive loads are applied, the 3D printing process leads the parts to show higher stiffness than in the IM process. In compression tests, this is due to the direction of the force being opposite to that in the tensile test and the separation of consecutive layers of the printed parts is more difficult. Thus, using high infill density values avoids an early breakage between consecutive layers. Qualitatively, it can be observed that the behaviour of the 3D printed part decreases as of a determined strain. Furthermore, the decline appears just when the compressive stresses of both samples are equal. This decrease is a consequence of the separation taking place between consecutive layers. This conclusion is supported by the enormous separation observed in Figure 10B corresponding to 60%-filled parts, while this occurred incrementally in completely filled samples. In the 60%-filled non-unidirectional samples (Figure 10C,D), polymeric hardening took place under compression stresses (Figure 11). This can be an advantage for parts that work under compressive stresses with no deformability constraints. Inversely, the unidirectional pattern broke down due to the presence of gaps between consecutive layers. Improvements up to 73% in yield strength and 33% in Young’s Modulus were reached when 100% instead of 60% infill density was used, as seen in Figure 9. 

It was possible to see a slight influence of infill pattern for yield strength (unidirectional had a higher yield strength than triangles, and these were higher than the ±45° alternate linear one). These smaller differences were due mainly to the stress direction in the test, which tends to compress samples. In this case, the bonding between different layers was not as important as in the tensile test, where the commitment of transmitting load between consecutive layers came into play, and its low failure strength was a determining factor for the properties. However, Young’s Modulus was strongly affected by pattern. Filament orientation was due to pattern choice, which acted as a determining factor to consider the preferential direction of the stresses in each test and, consequently, in each functional application. Both the tensile and compression tests justify this conclusion. 

## 4. Conclusions

This work describes a comparative analysis of 3D printing and injection moulding technologies to establish the influence of the process on the behaviour of parts made of short carbon-fibre-reinforced PA6. Both the 3D printing and IM processes led to breakage of fibres, making them shorter by up to 24%. The thermal environment has been shown to have no influence on the behaviour of 3D printed parts. 

Tensile tests of printed parts showed worse results than for the IM parts, but differences did not exceed 21% for either yield strength, tensile strength or Young’s Modulus. These results are consistent with those obtained by other authors, which validates the IM process used as a reference. A non-linear relationship between infill density and mechanical properties is supported. The unidirectional pattern is the best choice when lower densities are used.

Compared to tensile tests, compression tests revealed a more similar behaviour of 3D printed parts and IM parts (only 4% improvement). Printed samples had higher stiffness values than the IM parts. This phenomenon has not been reported by other researchers. The selection of pattern is a determinant in this case since a hardening effect with the strain appeared for those manufactured using a non-unidirectional pattern, but without reaching the best results. 

The comparison between the tensile and compression tests revealed that this reinforced polyamide did not behave the same under compressive and stretching loads, regardless of the manufacturing process. Consequently, as previously reported in the literature, it is crucial to analyse the application for which parts are designed. These outcomes are a novel contribution to the existing literature.

## Figures and Tables

**Table 1:** Mechanical properties obtained from the bending tests.

Build Plate Temperature	110 °C		60 °C		25 °C	
Mechanical Properties	Top	Bottom	Top	Bottom	Top	Bottom
$E_{f}\;\left(\mathrm{MPa}\right)$	2078.45	2147.71	2147.71	1873.63	2437.25	2404.65
$Y_{f}\;\left(\mathrm{MPa}\right)$	47.39	47.61	46.10	43.29	52.77	50.79
$\sigma_{fM}\;\left(\mathrm{MPa}\right)$	68.95	71.04	67.99	62.81	73.63	71.60
